# Antimicrobial Activity of *Piper gaudichaudianum* Kuntze and Its Synergism with Different Antibiotics

**DOI:** 10.3390/molecules16129925

**Published:** 2011-12-01

**Authors:** Mariele Caroline Marques Nogueira Puhl, Diógenes Aparício Garcia Cortez, Tânia Ueda-Nakamura, Celso Vataru Nakamura, Benedito Prado Dias Filho

**Affiliations:** 1 Programa de Pós-graduação em Ciências Farmacêuticas, Universidade Estadual de Maringá, PR CEP 87020-900, Brazil; Email: marielecaroline@hotmail.com (M.C.M.N.P.); 2 Departamento de Farmácia e Farmacologia, Universidade Estadual de Maringá, PR. CEP 87020-900, Brazil; Email: dagcortez@uem.br (D.A.G.C.); 3 Departamento de Ciências Básicas da Saúde, Universidade Estadual de Maringá, PR. CEP 87020-900 Brazil; Email: tunakamura@uem.br (T.U.N.); cvnakamura@uem.br (C.V.N.)

**Keywords:** *Piper gaudichaudianum*, chromone, prenylated derivatives, antimicrobial activity, synergism

## Abstract

One of the oldest forms of medical practice is the use of plants for the treatment and prevention of diseases that affect humans. We have studied the antimicrobial activity and synergism of *Piper gaudichaudianum* Kuntze with different antibiotics. The crude extract from the leaves of *P. gaudichaudianum* was submitted to chromatographic separation, resulting in five fractions. Fraction F3 contained a chromone (2,2-dimethyl-6-carboxycroman-4-one), and fraction F2 contained isomers that are prenylated derivatives of benzoic acid [4-hydroxy-(3',7'-dimethyl-1'-oxo-octa-*E*-2'-6'-dienyl)benzoic acid and 4-hydroxy-(3',7'-dimethyl-1'-oxo-octa-2'-*Z*-6'-dienyl) benzoic acid]. The chemical structures of both compounds were determined by analysis of ^1^H-NMR, ^13^C-NMR, COZY, DEPT, HMQC, and HMBC spectral data, and by comparison with data in the literature. The crude extract, fraction F2, and fraction F3 showed good activity against *Staphylococcus aureus*, *Bacillus subtilis,* and *Candida tropicalis*. The two benzoic acid derivatives only showed activity against *S. aureus *and *B. subtilis*. The bioauthographic analysis showed an inhibition zone only in fraction F2. Fractions F2 and F3 showed synergism in combination with ceftriaxone, tetracycline, and vancomycin. Morphological changes in form and structure were found by scanning electron microscopy in *S. aureus* treated with the combination of fraction F2 with vancomycin.

## 1. Introduction

Active ingredients derived from natural products are of great importance for the discovery of new centers, which serve as a model in semi-synthesis and total syntheses [1]. Of the drugs currently used worldwide, about one-third are derived from natural products or are semi-synthetic [2]. The family Piperaceae is predominantly tropical, and includes 5 to 8 genera and approximately 2,000 species. The family is represented in Brazil by about 500 species, occurring in forest areas, particularly in the Atlantic Forest [3]. Chemical studies on *Piper* species have resulted in the isolation of many biologically active natural products, such as pyrenes, lignans, neolignans, terpenes, propenylphenols, chalcones, flavones, benzopyrenes, chromenes, lactones, and amides [4,5].

Teixeira [6] carried out a phytochemical study of immature and mature fruits of *P. gaudichaudianum*, and isolated and identified five non-volatile components: 5,7-Dihydroxy flavanone, (pinocembrine, **1**), 2,2-dimethyl-6-carboxycroman-4-one methyl ester (**2**), 2-(2'-hydroxy-5'-methoxyphenyl)-*N*-(2''-methylpropenyl)-2-oxo-acetamide (**3**), (*E*)-4(3'-decenyl) phenol (gibbilimbol B, **4**), and 2,2-dimethyl-2H-1 benzopyran-6-carboxylate (**5**). Substances **2** and **3** showed inhibitory activity against the microorganisms *Enterococcus faecium*, *Micrococcus luteus*, and *Staphylococcus faecium*, and substance **4** showed activity against *Rhodococcus equi*.

Ethanol extracts of the stem and leaves of *P. gaudichaudianum* and *P. aduncum* have been evaluated for trypanocidal activity against the epimastigote form of the Y strain of *Trypanosoma cruzi*, revealing a pronounced effect (IC_50_ = 404 and 112.4 µg/mL, respectively). Both compounds are chromenes, and the prenylated forms were more powerful. The activity of compounds **1** and **2** was modified through hydrogenation reactions and esterification, which showed that the reverse acid is important for the activity of this class of substances, as well as the unsaturation of the benzopyrene subunit [7].

*S. aureus* is one of the most important pathogenic bacteria, causing a wide range of infections from local and usually superficial infections to extremely severe systemic ones [8]. *S. aureus* can also cause toxic infections [9]. Methicillin-resistant *S. aureus* (MRSA) is resistant to all beta-lactam antibiotics. Resistant pathogenic strains have been the main cause of nosocomial infections, causing considerable mortality of patients, and high health-care costs [10,11,12].

Antimicrobial combinations are employed in order to prevent the emergence of resistant strains or to increase activity, in cases of mixed infections or to reduce the toxicity of a substance without compromising the antimicrobial action. Interactions between different drugs may be synergistic, antagonistic, or indifferent.

Many studies have examined the synergism involving the action of plant derivatives against *S. aureus*. The porphyrin pheophorbide A and flavonolignan 5'-methoxyhydnocarpin (5'-MHC), isolated from plants of the genus *Berberis*, inhibit the efflux pump in *S. aureus*, thereby boosting the antibacterial alkaloid berberine [13]. Piperine, the alkaloid found in plants of the family Piperaceae, has synergistic activity in combination with ciprofloxacin against *S. aureus*, including MRSA strains [14]. Totarol, also a phenolic diterpene, showed activity in inhibiting the efflux pump of *S. aureus* [15]. The purpose of this study was to isolate the chemicals present in the leaves of *P. gaudichaudianum*, and to evaluate their antibacterial activity and synergism with other antibiotics against *S. aureus*.

## 2. Results and Discussion

**Figure 1:**
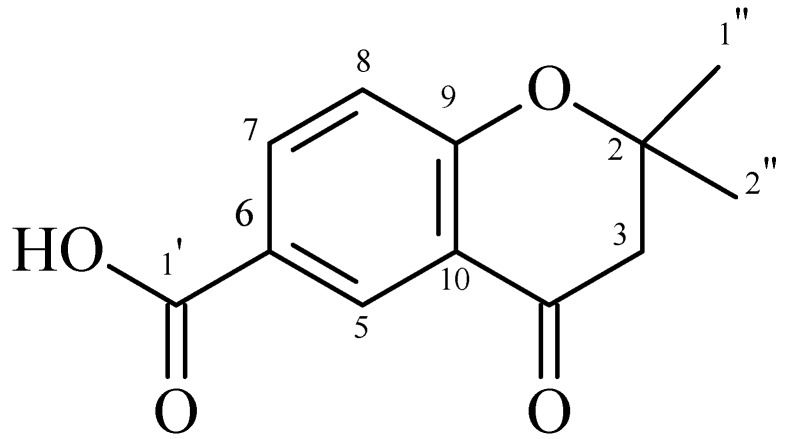
2,2-dimethyl-6-carboxycroman-4-one.

**Figure 2:**
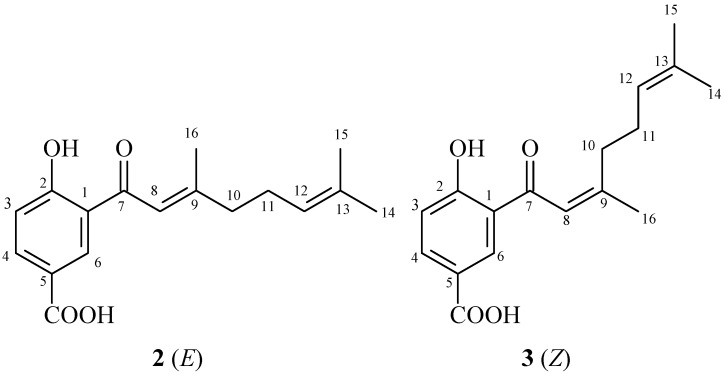
Structures of prenylated benzoic acid derivatives **2** and **3**.

The crude extract from the leaves of *P. gaudichaudianum* was fractionated, resulting in five fractions. Fraction F3 contained a chromone (2,2-dimethyl-6-carboxycroman-4-one) (Figure 1). Fraction F2 contained isomers that are prenylated derivatives of benzoic acid (4-hydroxy-(3',7'-dimethyl-1'-oxo-octa-*E*-2'-6'-dienyl) benzoic acid and 4-hydroxy-(3',7'-dimethyl-1'-oxo-octa-2'-*Z*-6'-dienyl) benzoic acid) (Figure 2). The chemical structures of both compounds were determined by analysis of ^1^H-NMR, ^13^C-NMR, COZY, DEPT, HMQC, and HMBC spectral data, and by comparison with values reported in the literature [6,16].

Although Teixeira [6] isolated a similar substance from fruits of *P. gaudichaudianum*, this is the first time that 2,2-dimethyl-6-carboxycroman-4-one has been isolated from the leaves of this species. The benzoic acid isomers were previously isolated from leaves of *Piper murrayanum* [17], and leaves of *Piper lhotzkyanum* [18]. Recently, 4-hydroxy-(3',7'-dimethyl-1'-oxo-octa-*E*-2'-6'-dienyl) benzoic acid was isolated and identified in a dichloromethane-methanol fraction obtained from the roots of *Piper crassinervium* [16]. Many benzoic acid derivatives have been isolated from species of Piperaceae and identified. This is the first report of this isomer in *P. gaudichaudianum*.

A previous report by Silva and co-workers [17] showed that the hydroalcoholic extract and the amides piperovatine and piperlonguminine from *P. ovatum *Vahl have good antimicrobial activity against *B. subtilis *and *C. tropicalis*. Another study reported the antibacterial activity of both crude extract and purified active compound of *Piper regnellii*, traditionally used in Brazilian folk medicine to treat infectious diseases [18] The compound that showed antibacterial activity against methicillin-resistant *S. aureus *(MRSA) was identified as eupomatenoid-5 by spectroscopic analysis.

In a 2010 study by Koroishi *et al.*, the *in vitro* antidermatophyte activity of the extracts and derivatives from leaves of *Piper regnellii *showed great antifungal activity against *Trichophyton rubrum*, and nail lacquer containing its chloroform fraction may have great potential to treat onychomycosis caused by these microorganisms [19]

**Figure 3:**
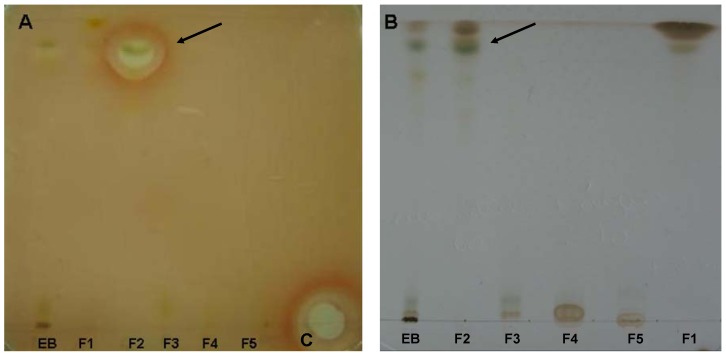
Bioautographic crude extracts and fractions isolated from the leaves of *P. gaudichaudianum*. The plate **A** was used for bioautography with *S. aureus* and the plate **B** was revealed with reactive Godin. Arrows indicate zones of inhibition of the fraction F2 and the band corresponding to the chromatographic plate. The reference compound on the plate was amikacin (**C**).

The crude extract and the fractions were tested against *S. aureus*, by bioautography (Figure 3). We observed a zone of inhibition in fraction F2 (dichloromethane-ethyl acetate, 95:5). The crude extract and other fractions showed no inhibition zones. The MICs of the aqueous extract, crude extract, fractions, and pure compounds tested against strains of *S. aureus*, *E. coli*, *Pseudomaonas aeruginosa*, *B. subtilis*, *Candida albicans*, *C. parapsilosis*, *C. tropicalis*, and *T. rubrum* by broth microdilution are presented in Table 1 and Table 2.

**Table 1:** Minimum inhibitory concentration (MIC), minimum bactericidal concentration (MBC) and minimum fungicidal concentration (MFC) of aqueous and crude extracts obtained from the leaves of *P. gaudichaudianum* and antibiotics used.

Samples/ Antibiotics	Antimicrobial Activity (µg/mL)															
	*S. aureus*		*B. subitilis*		*E. coli*		*P. aeruginosa*		*C. albicans*		*C. tropicalis*		*C. parapsilosis*		*T. rubrum*	
	MIC	MBC	MIC	MBC	MIC	MBC	MIC	MBC	MIC	MFC	MIC	MFC	MIC	MFC	MIC	MFC
AE	>1000	-	>1000	-	>1000	-	>1000	-	>1000	-	>1000	-	>1000	-	>1000	-
CE	250	500	62.5	62.5	>1000	-	>1000	-	>1000	-	62.5	125	>1000	-	1000	-
Ceftriaxone	6,25	-	-	-	-	-	-	-	-	-	-	-	-	-	-	-
Chloraphenicol	12,5	-	-	-	-	-	-	-	-	-	-	-	-	-	-	-
Penicillin	0,02	-	-	-	-	-	-	-	-	-	-	-	-	-	-	-
Tetracycline	0,39	-	-	-	1	-	12,5	-	-	-	-	-	-	-	-	-
Vancomycin	1,25	-	0,2	-	-	-	-	-	-	-	-	-	-	-	-	-
Fluconazole	-	-	-	-	-	-	-	-	7,8	-	>250	-	1,9	-	1,56	-

(−) unspecified.

**Table 2:** Minimum inhibitory concentration (MIC), minimum bactericidal concentration (MBC) and minimum fungicidal concentration (MFC) of fractions and pure compounds obtained from the leaves of *P. gaudichaudianum*.

Samples / Antibiotics	Antimicrobial Activity (µg/mL)															
	*S. aureus*		*B. subitilis*		*E. coli*		*P. aeruginosa*		*C. albicans*		*C. tropicalis*		*C. parapsilosis*		*T. rubrum*	
	MIC	MBC	MIC	MBC	MIC	MBC	MIC	MBC	MIC	MFC	MIC	MFC	MIC	MFC	MIC	MFC
F1	1000	-	1000	-	>1000	-	>1000	-	>1000	-	62.5	125	>1000	-	500	-
F2	62,5	62,5	15,6	15,6	>1000	-	>1000	-	>1000	-	62.5	125	>1000	-	250	-
F3	125	>1000	31.2	31.2	>1000	-	>1000	-	>1000	-	250	500	>1000	-	500	-
F4	>1000	-	>1000	-	>1000	-	>1000	-	>1000	-	500	500	>1000	-	1000	-
F5	>1000	-	>1000	-	>1000	-	>1000	-	>1000	-	1000	>1000	>1000	-	1000	-
FDA 47-58	31,25	62,5	7,81	7,81	>1000	-	>1000	-	>1000		1000	-	>1000	-	-	-
FDB 9	12,5	12,5	6,25	6,25	>1000	-	>1000	-	>1000	-	>1000	-	>1000	-	-	-

(−) unspecified.

The crude extract showed good activity against *B. subtilis* and *C. tropicalis* with MIC of 62.5 μg/mL, moderate activity against *S. aureus* with MIC of 250 μg/mL, weak activity against *T. rubrum* with MIC of 1,000 μg/mL, and had no effect on the other microorganisms. The aqueous extract proved to be inactive against the microorganisms tested. Fraction F1 (hexane) showed good activity against *C. tropicalis* with MIC of 62.5 μg/mL, moderate activity against *T. rubrum* with MIC of 500 μg/mL and was inactive against the other microorganisms.

Fraction F2 (dichloromethane-ethyl acetate 95:5) showed good activity against *B. subtilis*, *S. aureus*, and *C. tropicalis* with MICs of 15.6 μg/mL, 62.5 μg/mL, and 62.5 μg/mL, respectively; moderate activity against *T. rubrum* with MIC of 250 μg/mL; and was inactive against the other microorganisms.

Fraction F3 (dichloromethane-ethyl acetate 50:50) showed good activity against *B. subtilis* with MIC of 31.2 μg/mL, moderate activity against *S. aureus*, *C. tropicalis*, and *T. rubrum* with MIC 125 μg/mL, 250 μg/mL, and 500 μg/mL respectively; and was inactive against the other microorganisms.

Fraction F4 (ethyl acetate) showed moderate activity against *C. tropicalis* with MIC 500 μg/mL, and was inactive against the others. Fraction F5 (methanol) was inactive against all microorganisms tested.

Purified substances FDA 47-58 and FDB 9 showed activity against the bacteria *S. aureus* and *B. subtilis*, with MIC of 31.25 μg/mL and 12.5 μg/mL for *S. aureus*, respectively, and 7.81 μg/mL and 6.25 μg/mL for *B. subtilis* respectively.

Although the bioautography test showed an inhibition zone only against *S. aureus* for the F2 fraction, the minimum inhibitory concentration test showed that the EB, fraction F3 and F1, also possessed activity against *S. aureus* (Table 1 and Table 2). The low MICs could explain the absence of the inhibition of the other fractions and EB in the assays.

In general, secondary metabolites of plants are a source of bioactive substances, and scientific interest in these has been increasing during the search for new drugs, as well as new centers that can serve as model compounds. Chromones are not as abundant in *Piper* species as are amides, lignoids, phenylpropanoids and terpenes, but these substances are rich in pharmacological potential. Chromones in immature and mature fruits of *P. gaudichaudianum* show activity against *E. faecium*, *M. luteus*, and *S. faecium* [6]. Salazar *et al*. [16] found antifungal activity of chromones of leaves and stems of *Peperomia villipetiola* against *Cladosporium cladosporioides* and *C. sphaerospermun.* Chromones isolated from *Peperomia serpens* also showed antifungal activity [20]. Other chromones obtained through synthesis have shown activity against MRSA [21]. Chromones and chromanones isolated from a methanol extract of *Hypericum sikokumontanum* showed activity against *Helicobacter pylori* in a study by Tanaka *et al.* [22]. *Piper hostmannianum* and *Piper aduncum* yielded a benzoic-acid derivative 3-(2-hydroxy-3-methyl-3-butenyl)-4-methylhydroxybenzoate with molluscicidal activity [23,24]. From *Piper lanceaefolium,* prenylated derivatives of benzoic acid that had antifungal activity were isolated [25]. *Piper multiplinervium* accumulates a derivative of benzoic acid with antimicrobial activity against *S. aureus*, *E. coli*, *Klebisiella pneumoniae*, *Mycobacterium smegmatis*, *P. aeruginosa*, and *C. albicans* [26]. These studies indicate a tendency for chromones and derivatives of benzoic acid to show antimicrobial activity, as also found in this study (Table 2).

**Figure 4:**
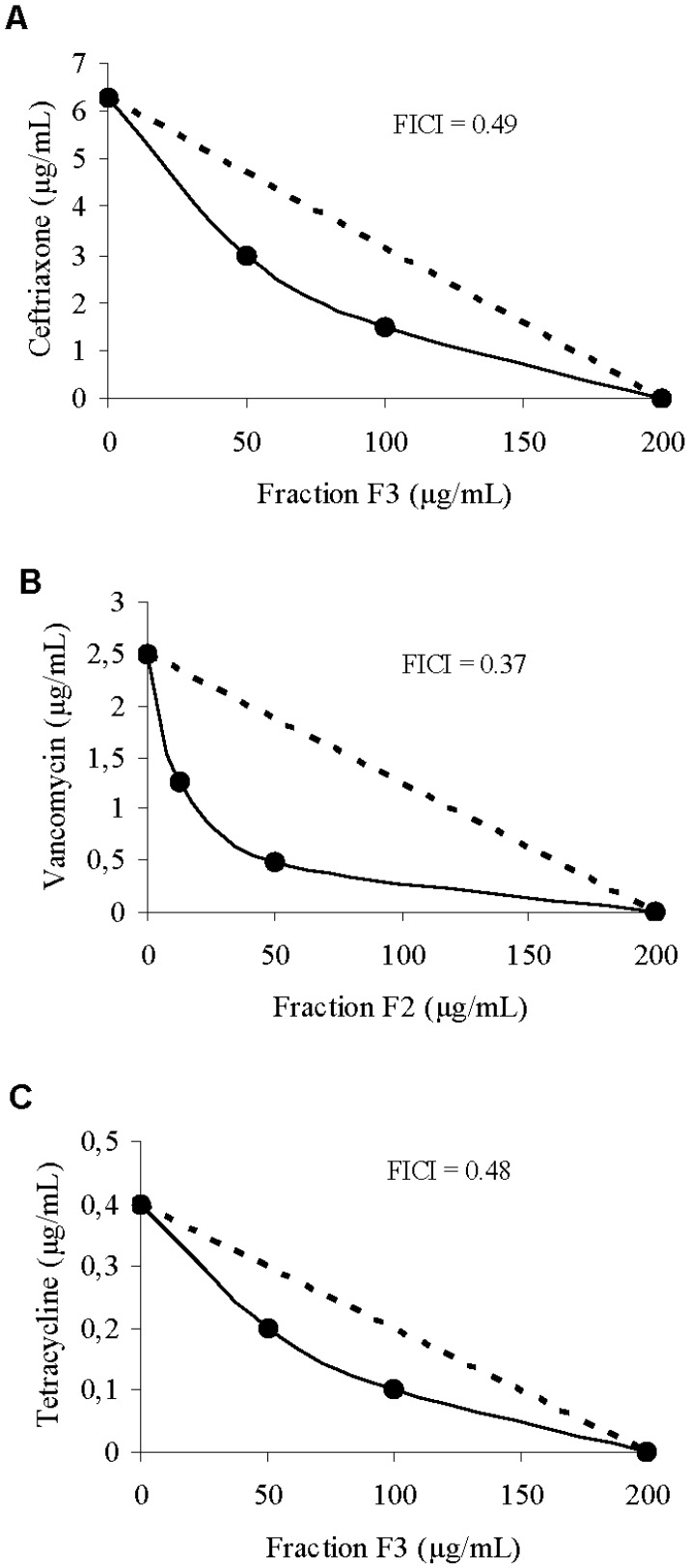
(**A**) Isobologram representing synergy between ceftriaxone and F3 against *S. aureus*; (**B**) Isobologram representing synergism between vancomycin and F2 against *S. aureus*; (**C**) Isobologram representing synergism between tetracycline and F3 against *S. aureus*; The dashed line indicates the theoretical additive activity.

The synergism of the crude extract, fraction F2, and ejection fraction F3 with antibiotics of different classes was verified through the checkerboard technique and the isobologram (Figure 4). The MIC of ceftriaxone was 6.25 μg/mL when the drug was tested alone, and decreased to 1.56 μg/mL in the presence of 100 μg/mL of fraction F3. For vancomycin, MIC was 1.25 μg/mL when tested alone, and decreased to 0.31 μg/mL in the presence of 50 μg/mL of fraction F2. 

For tetracycline, the MIC was 0.39 μg/mL when tested alone, and decreased to 0.09 μg/mL in the presence of 100 g/mL of fraction F3. Several studies of natural products have demonstrated the effect of the combination of drugs against microorganisms. Stermitz *et al*. [13] demonstrated the synergistic activity of porphyrin compounds and flavonolignans with the alkaloid berberine; the compounds inhibited the efflux pump of *S. aureus*, potentiating the antibacterial effect of berberine. Compounds such as diterpenes isolated from *Rosmarinus officinalis* and *Lycopus europaeus* showed a synergistic effect with erythromycin against resistant strains of *S. aureus* [27,28,29,30].

## 3. Experimental

### 3.1. General

The NMR spectra were obtained in a Bruker ARX400 (9.4 T) and Varian Gemini 300 (7.05 T) instruments, using deuterated solvent for field homogeneity, TMS as internal standard and temperature constant of 298 K. IR: film NaCl plates; ES-MS were recorded on a Micro-mass Quattro LC, HRMS: Auto-spec Micro-mass EBE and EI-MS on a CG/EM-SHIMADZU QP 2,000 A. CC: silica gel 60 (70–230 and 230–400 mesh); TCL: silica gel plates F254 (0.25 mm in thickness).

### 3.2. Plant Material

Leaves of *P. gaudichaudianum *were collected in August of 2008 in the Didactic Garden and Experimental Biology Department of the campus of the State University of Maringá. A voucher specimen (HUM 9616) was deposited in the herbarium of the Department of Botany of the State University of Maringá, Paraná.

### 3.3. Plant Extraction and Purification

The leaves (720 g) of the *P. gaudichaudianum* were extracted with ethanol-water (9:1 v/v, 7L) for 48 h at room temperature. The solvent was removed under vacuum at 40 °C to give an aqueous extract and a dark green residue. The aqueous extract from the crude hydroalcoholic extract was lyophilized (16.4 g) and the residue from crude extract in glass bottle was washed with dichloromethane. The organic solvent was removed to give the dichloromethane extract (11.87 g), which was chromatographed in a vacuum silica-gel apparatus, eluted with gradients of hexane, dichloromethane-ethyl acetate (95:5 v/v), dichloromethane-ethyl acetate (1:1 v/v), ethyl acetate and methanol, affording fractions F1 (0.44 g), F2 (2.72 g), F3 (1.18 g), F4 (0.7 g) and F5 (4.62 g). The dichloromethane-ethyl acetate (1:1 v/v) fraction F3 was rechromatographed on a silica gel 60 (70–230 mesh) column eluted with gradients of the hexane, hexane/EtOAc (95:5, 90:10, 80:20, 70:30, 60:40 and 50:50 v/v), EtOAc, EtOAc/MeOH (95:5, 75:25 and 50:50) and MeOH, affording 191 fractions. The combined subfractions 47–58 (29.70 mg) were identified as a chromone (2,2-dimethyl-6-carboxycroman-4-one). The dichloromethane-ethyl acetate (95:5 v/v) fraction F2 was rechromatographed on a silica gel 60 (70–230 mesh) column eluted with gradients of the hexane, hexane/EtOAc (80:20, 70:30, 60:40 and 50:50 v/v), EtOAc, and MeOH, affording 156 fractions. The subfractions 9 (17.50 mg) was identified as isomeric prenylated benzoic acid derivatives [4-hydroxy-(3',7'-dimethyl-1'-oxo-octa-*E*-2'-6'-dienil) benzoic acid and 4-hydroxy-(3',7'-dimethyl- 1'-oxo-octa-2'-*Z*-6'-dienyl) benzoic acid]. The chemical structures of the isolated substances were determined by ^1^H-NMR, R^13^C-NMR, COZY, DEPT, HMQC, HMBC spectral analysis of and by comparison with values reported in the literature [6,16].

*2,2-Dimethyl-6-carboxycroman-4-one*. The chemical structure was determined in comparison with values of NMR spectra in the literature [6], DEPT, HMQC, HMBC and by comparison with other literature data [6,16]. ^1^H-NMR (CDCl_3_, 300 MHz): δ 2.8 (d, *J* = 16.5 Hz, H-3a); 2.71 (d, *J* = 16.5 Hz, H-3b); 8.63 (d, *J* = 2.1 Hz, H-5); 8.18 (dd, *J* = 8.7 Hz and *J* = 2.1 Hz, H-7); 7.01 (d, *J* = 8.7 Hz, H-8); 1.58 (s, H1''); 1.58 (s, H2''). ^13^C-NMR (CDCl_3_, 75.50 MHz): δ 82.54 (C-2); 47.38 (C-3); 191.77 (C-4); 130.07 (C-5); 120.10 (C-6); 137.49 (C-7); 118.97 (C-8); 163.87 (C-9); 122.29 (C-10); 167.91 (C-1'); 25.88 (C-1'' and 2'').

*4-Hydroxy-(3',7'-dimethyl-1'-oxo-octa-E-2'-6'-dienyl) benzoic acid*. The chemical structure was determined in comparison with the NMR spectra values in the literature [16]. ^1^H-NMR (CDCl_3_, 300 MHz): δ 7.03 (d, *J* = 9 Hz, H-3); 8.15 (dd, *J* = 1.5 and 9 Hz, H-4); 8.56 (d, *J* = 1.8 Hz, H-6); 6.84 (s, H-8); 2.29–2.35 (m, H-10); 2.33–2.37 (m, H-11); 5.15–5.18 (m, H-12); 1.65 (s, H-14); 1.67 (s, H-15); 2.22 (s, H-16); 13.42 (s, OH). ^13^C-NMR (CDCl_3_, 75,50 MHz): δ 119.86 (C-1); 167.94 (C-2); 119.39 (C-3); 137.32 (C-4); 120.38 (C-5); 133.28 (C-6); 196.05 (C-7); 119.12 (C-8); 163.7 (C-9); 41.89 (C-10); 26.12 (C-11); 122.81 (C-12); 130.08 (C-13); 25.90 (C-14); 16.48 (C-15); 20.50 (C-16); 170.62 (COOH).

*4-Hydroxy-(3',7'-dimethyl-1'-oxo-octa-2'-Z-6'-dienyl) benzoic acid*. ^1^H-NMR (CDCl_3_, 300 MHz): δ 7.03 (*d*, *J* = 9 Hz, H-3); 8.15 (*dd*, *J = *1,5 and 9 Hz, H-4); 8.58 (*d*, *J* = 1.8 Hz, H-6); 6.84 (*s*, H-8); 2.01–2.1 (*m*, H-10); 2.29–2.31 (*m*, H-11); 5.15–5.18 (*m*, H-12); 1.65 (*s*, H-14); 1.64 (*s*, H-15); 2.24 (*s*, H-16); 13.42 (*s*, OH). ^13^C-NMR (CDCl_3_, 75,50 MHz): δ 119.86 (C-1); 167.94 (C-2); 119.39 (C-3); 137.32 (C-4); 120.38 (C-5); 133.28 (C-6); 196.05 (C-7); 119.12 (C-8); 164.32 (C-9); 35.75 (C-10); 26.12 (C-11); 122.81 (C-12); 130.08 (C-13); 25.90 (C-14); 16.48 (C-15); 26.13 (C-16); 170.62 (COOH).

### 3.4. Thin Layer Chromatography

Kieselgel GF_254_ plates, 20 × 20 cm, 1 mm thick, were used. Plant extracts (1 μg/mL) were applied (50 µL) and the chromatogram developed using hexane-ethyl acetate (50:50) as solvent. TLC plates were run in duplicate and one set was used as the reference chromatogram. Spots and bands were visualized by UV irradiation (245 and 366 nm) and Godin’s Reactive (solution of 1% of vanillin and perchloric acid in a 1:1 proportion). The other set was used for bioautography. Amikacin (12.8 µg, Bristol Myers Squibb) was used as reference antibiotic.

### 3.5. Strains and Growth Conditions

The test microorganisms used included *Staphylococcus aureus* ATCC 25923, *Escherichia coli* ATCC 25922, *Pseudomonas aeruginosa* ATCC 27853 and *Bacillus subtilis* ATCC 6623, *Candida albicans* ATCC 10231, *Candida parapsilolis* ATCC 22019, *C. tropicalis* ATCC 28707, *Trichophyton rubrum *ATCC 28189. 

Bacteria were maintained on Mueller Hinton Agar and sub cultured in Mueller Hinton Broth before each experiment. Yeasts were maintained at 4 °C on Sabouraud Dextrose Agar plates and sub cultured at 37 °C in Sabouraud Dextrose Broth before each experiment, to ensure viability and purity. The dermatophyte, was maintained on Sabouraud Dextrose Agar slants at 10 °C and sub cultured monthly throughout this study.

### 3.6. Bioautography

Chromatograms developed as described above were placed in a square plate with cover and inoculums of *S. aureus *containing 10^6^ CFU/mL in molten Mueller-Hinton agar was distributed over the place. After solidification of the medium, the TLC plate was incubated overnight at 37 °C. Subsequently the bioautogram was sprayed with an aqueous solution of 2,3,5-triphenyltetrazolium chloride (TTC) and incubated at 37 °C for 4 h. Inhibition zones indicated the presence of active compounds.

### 3.7. Antibacterial Susceptibility Testing

The minimum inhibitory concentrations (MICs) of all compounds and reference antibiotics were determined by micro dilution techniques in Mueller Hinton broth for bacteria, and Sabouraud broth for yeasts, described by the Clinical and Laboratory Standards Institute (CLSI) [34]. Inoculates were prepared in the same medium at a density adjusted to a 0.5 McFarland turbidity standard (10^8^ colony-forming units [CFU]/mL) and diluted 1:10 for the broth micro dilution procedure. Micro titer trays were incubated at 37 °C, and the MICs were recorded after 24 h for bacteria and 48 h for yeast of incubation. For dermatophyte the period of incubation time was 72 h at 28 °C. Two susceptibility endpoints were recorded for each isolate. The MIC was defined as the lowest concentration of compounds at which the microorganism tested did not demonstrate visible growth. Minimum bactericidal concentration (MBC) and Minimum fungicidal concentration (MFC) were defined as the lowest concentration yielding negative subcultures or only one colony.

### 3.8. Scanning Electron Microscopy

**Figure 5:**
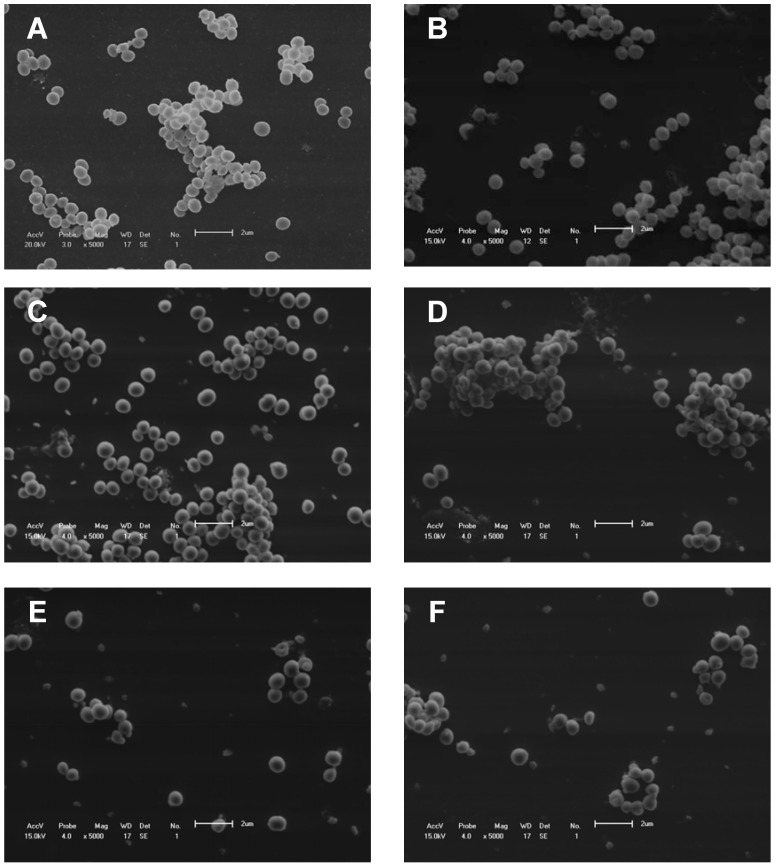
Scanning electron microscopy. (**A**) *S*. aureus without treatment (control); (**B**) *S*. aureus treated with fraction F2 (50 mg/mL); (**C** and **D**) *S.* aureus treated with vancomycin (0.63 μg/mL). (**E** and **F**) *S.* aureus treated with combinations of fraction F2 (6.25 μg/mL) and vancomycin (0.63 μg/mL).

To investigate the effect of fraction F2 and vancomycin alone and in combination on the morphology of *S. aureus*, treated and control cells were examined by scanning electron microscopy. Cells were fixed with 2.5% glutaraldehyde in 0.1 M cacodylate buffer, pH 7.2, and small drops of the fixed cells were placed on a specimen support with poly-L-lysine for 1 h at room temperature. Subsequently, the samples were dehydrated in a graded ethanol series, critical-point dried in CO_2_, coated with gold, and examined under a Shimadzu SS-550 scanning electron microscope (Figure 5).

### 3.9. Checkerboard

From the results of the minimum inhibitory concentration tests, the combination of EB, F2, and F3 was done with the antibiotic ceftriaxone, chloramphenicol, penicillin, tetracycline, and vancomycin using the “checkerboard” method against the bacterium *S. aureus*. The combinations of EB and fractions with the antibiotics were tested in Mueller-Hinton Broth with an inoculum of 1 × 10^4^ CFU/mL of *S. aureus* and different concentrations of the drug, ranging from 500 to 3.13 µg/mL, and antibiotics, ranging from 25 to 0.01 µg/mL.

In a 96-well plate, each well received 100 μL of HCM, except column 12A, which received 200 μL of the antibiotic to be tested. The remaining wells of column 12 received 100 μL of each antibiotic, followed by serial dilutions to column 2. Then, the wells of the line received 100 μL of EB or fractions, followed by serial dilutions up to row G. Row H and column 1 were the controls for the antibiotic and EB/fractions, respectively. Each well contained a combination of different concentrations of EB and fractions with antibiotics. The plates were incubated at 37 °C for 24 h, and the results were assessed by observing the inhibition of visible growth [35,36]. The combinations of the substances were analyzed by calculating the FIC index (FICI) as follows: FIC = (MICa of the combination/MICa alone) + (MICb of the combination/MICb alone). The FIC was interpreted as: (i) a synergistic effect when ≤0.5; (ii) an additive or indifferent effect when >0.5 and ≤4; and (iii) an antagonistic effect when >4 [37]. The combination of the two components is shown graphically by a Cartesian diagram, by applying the isobole method. The non-interaction of the two components results in a straight line, whereas the occurrence of an interaction is shown by a concave isobole [38].

## 4. Conclusions

The results of this study of the antimicrobial properties of fractions and mainly of pure substances, together with their synergistic activity, is promising from the standpoint of medicinal chemistry, in the search for bioactive compounds from plants that may provide prototype molecules for the synthesis of more potent, selective, less-toxic and low-cost analogues. However we need to carry out further research to elucidate the mechanism of action of synergistic components, as well as their applications as viable antimicrobial agents.
